# The association between mental illness and all-cause mortality in patients with cirrhosis: a Veterans Affairs retrospective cohort study

**DOI:** 10.1097/HC9.0000000000000129

**Published:** 2023-03-30

**Authors:** Lauren R. Shaffer, David E. Kaplan, Tamar H. Taddei, Nadim Mahmud

**Affiliations:** 1 Department of Medicine, University of Pennsylvania Perelman School of Medicine, Philadelphia, Pennsylvania, USA; 2 Division of Gastroenterology and Hepatology, University of Pennsylvania Perelman School of Medicine, Philadelphia, Pennsylvania, USA; 3 Gastroenterology Section, Corporal Michael J. Crescenz VA Medical Center, Philadelphia, Pennsylvania, USA; 4 Division of Digestive Diseases, Yale University School of Medicine, New Haven, Connecticut, USA; 5 VA Connecticut Healthcare System, West Haven, Connecticut, USA; 6 Leonard David Institute of Health Economics, University of Pennsylvania Perelman School of Medicine, Philadelphia, Pennsylvania, USA; 7 Department of Biostatistics, Epidemiology, and Informatics, University of Pennsylvania, Philadelphia, Pennsylvania, USA

## Abstract

Mental health diagnoses are common and known to impact key outcomes in patients with chronic illnesses including cirrhosis. However, the independent impact of psychiatric comorbidities on mortality in these patients and potential mitigating effects of outpatient mental health–related care has not been well characterized.

**Methods:**

This was a retrospective cohort study of patients with cirrhosis in the Veterans Health Administration between 2008 and 2021. Adjusted Cox regression was performed to evaluate the association between mental health–related diagnoses [groups: alcohol use disorder (AUD)/substance use disorder (SUD) alone, non-AUD/SUD alone, and any mental health diagnosis (AUD/SUD or non-AUD/SUD)] and all-cause mortality. In subgroup analyses, the impact of regular outpatient mental health visits was also assessed.

**Results:**

We identified 115,409 patients, 81.7% of whom had any mental health diagnosis at baseline. During the study window there was a significant increase in the number of mental health clinic visits per person-year (β=0.078, 95% CI: 0.065–0.092, *p* < 0.001), but a decrease in AUD/SUD clinic utilization (*p* < 0.001). In regression models, there was a 54% increased hazard in all-cause mortality for any mental health diagnosis, 11% for non-AUD/SUD, and 44% for AUD/SUD (each *p* < 0.001). Regular mental health visits resulted in a 21% decreased risk in all-cause mortality for AUD/SUD diagnosis, compared with 3% and 9% for any mental health diagnosis and non-AUD/SUD diagnosis, respectively (each *p* < 0.001).

**Conclusions:**

Mental illness is associated with an increased risk of all-cause mortality in veterans with cirrhosis. Regular outpatient mental health care may be protective against all-cause mortality, particularly among patients with AUD/SUD. Future studies should focus on relevant clinical practice changes, including implementing integrated care programs.

## Graphical abstract

**Figure:**
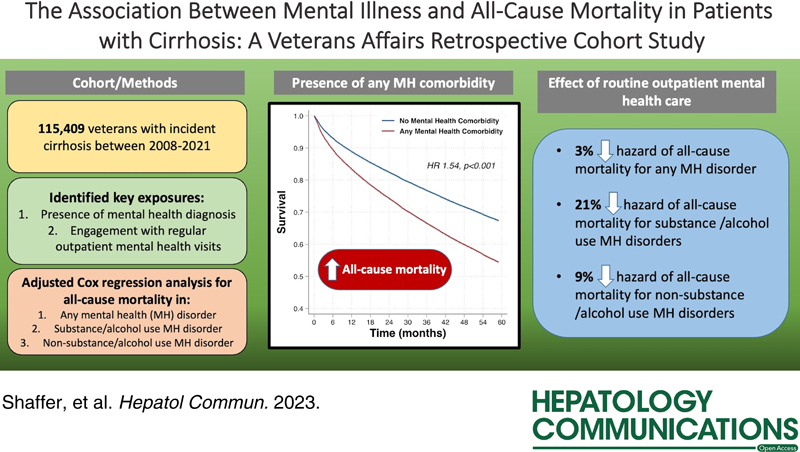

## INTRODUCTION

Mental illness is commonly underdiagnosed and undertreated in individuals with chronic diseases, including chronic liver disease (CLD). Unsurprisingly, the burden of mental health comorbidities, such as depression which has been commonly studied, is higher in patients with CLD than in the general population.^1^,^2^ Many patients with CLD also knowingly suffer from substance use disorder (SUD), of which alcohol use disorder (AUD) remains one of the leading causes of cirrhosis.^3^ While patients may suffer independently from SUD, they are also prone to having concomitant psychiatric disease impacting their care.^3^ Ultimately, comorbid mental illness in CLD patients has been associated with worsened disease outcomes, including decreased quality of life, reduced engagement in care, worsened cognitive function, frailty, and increased mortality.^2^,^4^–^6^ This demonstrates the urgency of characterizing the contemporary burden of mental health illness in patients with cirrhosis, and comprehensively understanding the link between mental illness and key outcomes in these patients.

While the broad effect of mental illness has been shown to negatively impact an array of patient outcomes, the potential effect of outpatient mental health care services in ameliorating such harms is less well characterized. One promising approach that has been well-studied and widely accepted in chronic illness management is an integrated care model, which involves strategic coordination in delivering comprehensive health and social care services to patients using a multidisciplinary approach.^7^ This approach has had favorable outcomes in patients with chronic illnesses such as chronic obstructive pulmonary disease and congestive heart failure.^4^,^8^,^9^ Integrated care models have also recently gained traction in managing patients with specific liver diseases, with prior studies investigating the role of multidisciplinary care in patients with fatty liver disease, HCC, HCV infection, and alcohol-associated liver disease (ALD).^4^,^10^–^14^ The utility of this model may be further expanded to patients specifically with cirrhosis and comorbid mental illness, however, the impact of routine outpatient mental care on outcomes has not been studied in a large cohort to date.

US veterans represent a unique cohort that may provide insight into the relationship between cirrhosis, mental illness, and mortality due to a high burden of psychiatric and substance use–related comorbidities. In this study, we used robust national data from the Veterans Health Administration (VHA) in patients with cirrhosis to evaluate: (1) temporal trends in mental health disorders and associated outpatient care, (2) the association between different classes of mental health comorbidities and all-cause mortality, and (3) whether engagement with regular outpatient mental health care impacts this association.

## METHODS

### Study design and data source

This study was a retrospective cohort study utilizing a well-established database of patients with cirrhosis followed across 128 centers in the US VHA. This cohort entitled VOCAL (Veterans Outcomes and Costs Associated with Liver Disease) contains extensive longitudinal data from over 125,000 veteran patients with cirrhosis identified in the VHA between January 1, 2008, and December 31, 2021, and has been extensively utilized for natural history studies of patients with cirrhosis.^15^–^17^ In this study, we included patients age 18 years and above with incident cirrhosis diagnosis (index date), identified following previously validated algorithms.^18^ We excluded patients with prior liver transplantation, HCC at index, or <30 days of follow-up.

### Ascertainment of exposures

For each patient, we obtained demographic information, body mass index, Alcohol Use Disorders Identification Test-Concise (AUDIT-C), laboratory results and Model for End-stage Liver Disease-Sodium (MELD-Na) at index, and comorbidities (diabetes, coronary artery disease, heart failure, atrial fibrillation). Etiology of liver disease was ascertained according to the VHA algorithm from Beste et al^19^ and classified as HCV, HBV, ALD, HCV+ALD, NAFLD, or other causes. Child-Turcotte-Pugh class and prior decompensated cirrhosis were also determined according to previously validated algorithms in the VHA.^20^,^21^ The primary exposures were mental health diagnoses of interest; these were identified using outpatient and inpatient International Classification of Diseases (ICD)-9 and ICD-10 codes following previously validated approaches where available.^22^–^26^ Specific diagnoses included depression, anxiety disorder, posttraumatic stress disorder (PTSD), bipolar disorder, schizophrenia, AUD, and SUD (Supplemental Table 1, http://links.lww.com/HC9/A234). Outpatient visits providing mental health–related care were identified using primary and secondary clinic stop codes, which are 3 digit designations that identify the main clinical outpatient group responsible for a particular type of care (Supplemental Table 2, http://links.lww.com/HC9/A234).^27^ Based on stop code designations, these visits were categorized as mental health clinic/primary care, AUD/SUD-related visit, or other (Supplemental Table 2, http://links.lww.com/HC9/A234). Importantly, all mental health diagnoses of interest were obtained in time-updated fashion (updated every 30 d), from baseline (time of cirrhosis diagnosis) through maximum follow-up (December 31, 2021). Similarly, all outpatient mental health–related visits were obtained for the cohort through maximum follow-up.

### Primary outcome

The primary outcome of interest was time to all-cause mortality. This was ascertained using the Vital Status File, which is a well-validated source of death data in the VHA.^28^

### Statistical analysis

Descriptive statistics were presented as medians and interquartile ranges for continuous variables and counts and percentages for categorical variables. Data were stratified by the following baseline categories: (1) non-AUD/SUD mental health diagnosis (ie, depression, anxiety disorder, PTSD, bipolar disorder, schizophrenia), (2) AUD/SUD diagnosis, (3) both non-AUD/SUD and AUD/SUD diagnoses, and (4) no mental health–related diagnoses. Data were also presented as stratified by no mental health diagnosis versus any mental health diagnosis. Continuous variables were compared using the Kruskal-Wallis or Wilcoxon rank-sum tests, as indicated, and categorical variables with chi-square tests.

To evaluate trends in mental health diagnoses in the VHA cirrhosis cohort over time, we determined the number of patients at risk in each cohort year from 2008 to 2021 and computed the prevalence of each mental health diagnosis over time, in addition to the number of mental health visits per person-year. We also computed the incidence proportion in a subcohort limited to the post-ICD-10 era (2016 and beyond) due to known gaps in coding rates during the ICD-9/10 transition for several mental health conditions.^29^ Statistical significance of trends was determined using simple linear regression. Finally, to identify potential difference in prevalence of mental health diagnoses and outpatient visits by etiology of liver disease, we performed separate exploratory analyses for (1) HCV, (2) NAFLD, and (3) ALD patients.

To evaluate the potential association between mental health–related diagnoses on all-cause mortality, we used a survival analysis approach. To achieve balance in covariates at baseline (before regression modeling) we used an inverse probability treatment weighting (IPTW) approach. We first constructed a propensity score (PS) for mental health comorbidity using logistic regression models containing the following covariates: age, sex, race, body mass index, etiology of liver disease, diabetes, coronary artery disease, heart failure, atrial fibrillation, prior cirrhosis decompensation, MELD-Na, and AUDIT-C score. IPTWs were then calculated as 1/PS for patients with mental health comorbidities and as 1/(1−PS) for those without mental health comorbidity.^30^ Standardized mean differences were computed for raw and IPTW samples, with an absolute standardized mean difference <0.1 regarded to represent excellent covariate balance, consistent with best practice recommendations.^31^ Cox regression analysis with observations weighted by the IPTWs was then performed, where time zero was the date of cirrhosis diagnosis (index date) and observations were right-censored at maximum follow-up. Importantly, IPTW adjustment creates a pseudopopultaion where covariate balance is generally achieved without the need for additional covariate adjustment in regression models unless imbalance remains in the IPTW-adjusted sample. Three sequential models were performed with varying exposures: (M1) baseline mental health comorbidity, (M2) time-updating mental health comorbidity status (updated every 30 d, to account for patients who were diagnosed with mental health comorbidities during the course of follow-up), and (M3) subgroup analysis of patients with time-updating mental health comorbidities evaluating the impact of regular mental health outpatient care on the outcome. For the latter model, patients were considered be within regular outpatient care for 6 months after each outpatient visit, which as noted previously were obtained through maximum follow-up. Importantly, for each of the 3 models above, we performed 3 series of analyses where the mental health comorbidities of interest were varied. This included (S1) any mental health comorbidity (non-AUD/SUD or AUD/SUD), (S2) only non-AUD/SUD, and (S3) only AUD/SUD. In the latter 2 approaches, IPTWs incorporated the excluded mental health comorbidities to ensure balance in these conditions, which were also included as time-updating variables in models. Likewise, in subgroup analyses for these models (M3: ie, only including those with the mental health comorbidities of interest), the mental health visits defining regular outpatient care were limited to those addressing the conditions of interest. For example, only AUD/SUD outpatient visits were evaluated in models where AUD/SUD comorbidities were treated as the mental health comorbidities of interest. For all models, HRs and 95% CIs were reported. Adjusted survival curves were provided for all M2 models. An α threshold of 5% was used to determine statistical significance.

### Sensitivity analysis

Although we accounted for several key comorbidities through the IPTW procedure, we performed a sensitivity analysis in the primary all-cause models above (M1 and M2) where we additionally adjusted for the cirrhosis comorbidity score (CIRCOM).^32^ Finally, to address the possibility of healthy user bias—that patients completing outpatient mental health care visits may be generally more engaged in their health care—we repeated the above primary analyses in a subcohort limited to patients who had at least 2 outpatient visits in the index year.

### Exploratory analysis

To evaluate potential differences in cause of death, we performed a cause-specific mortality analysis where mortality events were considered to be liver-related if death was preceded by decompensation or HCC, and non–liver-related if mortality events occurred in the absence of prior decompensation or HCC. In IPTW-adjusted Cox regression models accounting for time-updated mental health comorbidities (any), we fit cause-specific competing risks regression models. Cause-specific HRs and 95% CIs were presented for both liver-related and non–liver-related mortality.

### Other considerations

This study received institutional review board approval from the Corporal Michael J. Crescenz Philadelphia Veterans Affairs Medical Center with a waiver of informed consent. Research was conducted in accordance with both the Declarations of Helsinki and Istanbul.

## RESULTS

### Cohort characteristics and mental health diagnosis

After applying exclusion criteria (Supplemental Figure 1, http://links.lww.com/HC9/A234), 115,409 patients with incident cirrhosis were included, 94,278 of whom (81.7%) had any mental health diagnosis (Supplemental Table 3, http://links.lww.com/HC9/A234). In the analytic cohort, 14,420 patients (12.5%) had a non-AUD/SUD diagnosis, 25,272 patients (21.9%) had an AUD/SUD diagnosis, and 54,586 patients (47.3%) had both AUD/SUD and non-AUD/SUD diagnoses (Table 1). Compared with 21,131 patients (18.3%) who had no mental health diagnosis, those with any mental health diagnosis tended to be younger (62 vs. 66, *p* < 0.001) and a higher proportion were Black (19.6% vs. 12.9%, *p* < 0.001; Supplemental Table 3, http://links.lww.com/HC9/A234). Comparing patients with no mental health diagnosis to other subcategories, individuals with AUD/SUD diagnosis alone or both AUD/SUD and non-AUD/SUD were more likely to have ALD (44.9% and 37.5% vs. 19.8%, respectively, *p* < 0.001) or HCV plus ALD (19.9% or 28.1% vs. 6.9%, respectively, *p* < 0.001), while patients with a non-AUD/SUD diagnosis alone were more likely to have NAFLD (53.4% vs. 41.7%, *p* < 0.001). For major comorbidities (ie, diabetes mellitus, coronary artery disease, heart failure, and atrial fibrillation), patients with a non-AUD/SUD diagnosis had higher rates of comorbidities as compared with all other subcategories (each *p* < 0.001). Finally, patients with AUD/SUD diagnosis alone at baseline were more likely to have decompensated cirrhosis compared with patients in other categories (*p* < 0.001; Table 1).

**TABLE 1:** Baseline cohort characteristics, stratified by MH diagnosis subcategories

	n (%)				
Factors	No MH diagnosis (N=21,131)	Non-AUD/SUD MH diagnosis (N=14,420)	AUD/SUD MH diagnosis (N=25,272)	Both AUD/SUD and non-AUD/SUD MH diagnoses (N=54,586)	*p*
Age [median (IQR)]	66 (60, 73)	66 (60, 72)	62 (57, 68)	62 (56, 66)	<0.001
Male sex	20,641 (97.7)	13,642 (94.6)	24,903 (98.5)	52,739 (96.6)	<0.001
Race					<0.001
White	14,162 (67.0)	9519 (66.0)	15,631 (61.9)	32,118 (58.8)	
Black	2730 (12.9)	1881 (13.0)	4842 (19.2)	11,748 (21.5)	
Hispanic	1710 (8.1)	1309 (9.1)	1836 (7.3)	4500 (8.2)	
Asian	341 (1.6)	259 (1.8)	283 (1.1)	636 (1.2)	
Other	2188 (10.4)	1452 (10.1)	2680 (10.6)	5584 (10.2)	
BMI [median (IQR)]	29.6 (26.0, 34.0)	30.9 (27.0, 35.5)	27.6 (24.0, 31.6)	28.0 (24.4, 32.3)	<0.001
Etiology of liver disease					<0.001
HCB	4083 (19.4)	3131 (21.8)	4289 (17.0)	10,633 (19.5)	
HBV	341 (1.6)	313 (2.2)	164 (0.6)	320 (0.6)	
ALD	4160 (19.8)	1866 (13.0)	11,336 (44.9)	20,484 (37.5)	
HCV+ALD	1449 (6.9)	709 (4.9)	5027 (19.9)	15,320 (28.1)	
NAFLD	8758 (41.7)	7677 (53.4)	3763 (14.9)	7283 (13.3)	
Other	2222 (10.6)	693 (4.8)	673 (2.7)	536 (1.0)	
Diabetes mellitus	11,414 (54.0)	9799 (68.0)	10,981 (43.5)	28,784 (52.7)	<0.001
Coronary artery disease	5308 (25.1)	5170 (35.9)	5112 (20.2)	14,090 (25.8)	<0.001
Heart failure	3616 (17.1)	3203 (22.2)	3753 (14.9)	8473 (15.5)	<0.001
Atrial fibrillation	3019 (14.3)	2390 (16.6)	2805 (11.1)	5528 (10.1)	<0.001
CTP class					<0.001
A	13,300 (62.9)	10,027 (69.5)	14,113 (55.8)	35,072 (64.3)	
B	6725 (31.8)	3965 (27.5)	9069 (35.9)	16,282 (29.8)	
C	1106 (5.2)	428 (3.0)	2090 (8.3)	3232 (5.9)	
Decompensated cirrhosis	4875 (23.1)	3206 (22.2)	6596 (26.1)	11,625 (21.3)	<0.001
TIPS	22 (0.1)	24 (0.2)	15 (0.1)	40 (0.1)	0.002
Sodium [median (IQR)]	138 (136, 140)	138 (136, 140)	137 (135, 140)	138 (135, 140)	<0.001
Creatinine [median (IQR)]	1.0 (0.8, 1.4)	1.0 (0.8, 1.4)	0.9 (0.8, 1.2)	0.9 (0.8, 1.2)	<0.001
Albumin [median (IQR)]	3.5 (3.0, 4.0)	3.6 (3.1, 4.0)	3.4 (2.8, 3.9)	3.5 (2.9, 4.0)	<0.001
Total bilirubin [median (IQR)]	1.0 (0.7, 1.7)	0.9 (0.6, 1.3)	1.1 (0.7, 1.9)	0.9 (0.6, 1.6)	<0.001
ALP [median (IQR)]	98 (73, 140)	96 (72, 134)	106 (79, 150)	102 (76, 143)	<0.001
AST [median (IQR)]	45 (29, 72)	41 (27, 65)	56 (32, 95)	54 (32, 96)	<0.001
ALT [median (IQR)]	36 (23, 62)	36 (23, 61)	38 (24, 65)	41 (24, 70)	<0.001
Platelet count [median (IQR)]	131 (92, 181)	138 (99, 188)	139 (98, 195)	144 (101, 200)	<0.001
INR [median (IQR)]	1.2 (1.1, 1.4)	1.2 (1.1, 1.4)	1.2 (1.1, 1.4)	1.2 (1.1, 1.4)	<0.001
MELD-Na [median (IQR)]	10 (6, 16)	9 (6, 14)	11 (7, 16)	9 (6, 15)	<0.001
HDL [median (IQR)]	40 (32, 52)	40 (32, 50)	41 (30, 55)	41 (31, 55)	<0.001
TGL [median (IQR)]	102 (73, 149)	116 (81, 173)	100 (73, 144)	109 (78, 159)	<0.001
LDL [median (IQR)]	80 (60, 103)	80 (60, 103)	83 (62, 106)	83 (62, 108)	<0.001
Total cholesterol [median (IQR)]	146 (120, 174)	147 (122, 176)	150 (122, 179)	152 (125, 182)	<0.001

Abbreviations: ALD, alcohol-associated liver disease; ALP, alkaline phosphatase; ALT, alanine transaminase; AST, aspartate transaminase; AUD, alcohol use disorder; BMI, body mass index; CTP, Child-Turcotte-Pugh; INR, international normalized ratio; MELD-Na, Model for End-stage Liver Disease-Sodium; MH, mental health; IQR, interquartile range; SUD, substance use disorder; TGL, triglyceride.

### Trends in mental health diagnoses and utilization of outpatient mental health care

**FIGURE 1:**
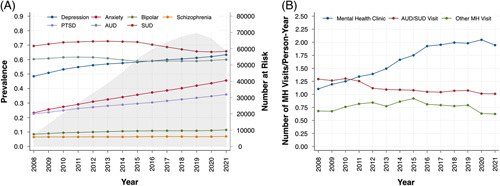
Trends in prevalence of MH diagnoses (A) and utilization of outpatient mental health care (B). Abbreviations: AUD, alcohol use disorder; MH, mental health; PTSD, posttraumatic stress disorder; SUD, substance use disorder.

Trends in prevalence of specific mental health diagnoses (ie, depression, anxiety, bipolar disorder, schizophrenia, PTSD, AUD, and SUD) over the period of 2008–2021 revealed that SUD, AUD, and depression had the highest prevalence, followed by anxiety and PTSD. In linear regression models, there were statistically significant increases in the prevalence of all non-AUD/SUD mental health diagnoses, most notably anxiety (β=0.017, 95% CI: 0.016–0.017, *p* < 0.001), depression (β=0.010, 95% CI: 0.008–0.011, *p* < 0.001), and PTSD (β=0.010, 95% CI: 0.009–0.010, *p* < 0.001; Figure 1A). The prevalence of AUD and SUD declined slightly over time (β=−0.002 and −0.005, respectively; each *p* < 0.05). However, the incidence proportions of all individual mental health diagnoses significantly increased since 2016 (β > 0, each *p*< 0.05), with AUD and SUD representing the highest incidence proportions overall (Supplemental Figure 2, http://links.lww.com/HC9/A234). Regarding engagement with outpatient mental health–related care, there was a significant increase in the number of visits per person-year for mental health clinic visits (β=0.078, 95% CI: 0.065–0.092, *p* < 0.001), whereas there was a gradual decrease in AUD/SUD clinic visits (β=−0.023, 95% CI: −0.030 to −0.016, *p* < 0.001; Figure 1B). Finally, in exploratory analyses, SUD was the most prevalent mental health diagnosis in HCV patients, whereas depression was more prevalent in NAFLD patients (Supplemental Figure 3, http://links.lww.com/HC9/A234). ALD patients had very high prevalence of AUD and SUD, as well as depression. Patients with ALD had the most rapid rate of rise in mental health clinic visits over time (β=0.138, 95% CI: 0.119–0.156, *p* < 0.001), followed by NAFLD patients (β=0.074, 95% CI: 0.067–0.081, *p* < 0.001) and then HCV patients (β=0.025, 95% CI: 0.017–0.034, *p* < 0.001; Supplemental Figure 3, http://links.lww.com/HC9/A234). Of note, despite rising prevalence of AUD (β=0.009, 95% CI: 0.008−0.009, *p* < 0.001) and SUD (β=0.004, 95% CI: 0.002–0.006, *p*=0.002) in patients with HCV cirrhosis, this group had a declining rate of AUD/SUD outpatient visits (β=−0.025, 95% CI: −0.036 to −0.015, *p* < 0.001).

### Association of any mental health diagnosis with all-cause mortality

**FIGURE 2:**
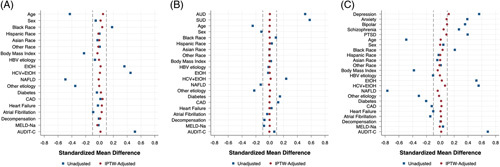
Covariate balance achieved through IPTW in analyses of any mental health comorbidity (A), non-AUD/SUD mental health comorbidities (B), and AUD/SUD mental health comorbidities (C). Abbreviations: AUD, alcohol use disorder; AUDIT-C, Alcohol Use Disorders Identification Test-Concise; CAD, coronary artery disease; IPTW, inverse probability treatment weighting; MELD-Na, Model for End-stage Liver Disease-Sodium; SUD, substance use disorder.

**FIGURE 3:**
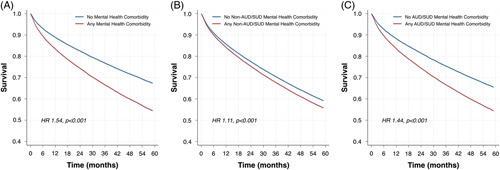
Inverse probability treatment weighting Cox-adjusted survival curves for analyses of any mental health comorbidity (A), non-AUD/SUD mental health comorbidities (B), and AUD/SUD mental health comorbidities (C). Abbreviations: AUD, alcohol use disorder; SUD, substance use disorder.

To account for differences in cohort characteristics among covariates of age, sex, race, body mass index, etiology of liver disease, diabetes mellitus, coronary artery disease, congestive heart failure, atrial fibrillation, prior cirrhosis decompensation, MELD-Na, and AUDIT-C score, IPTW adjustment based on PS for any mental health diagnosis was conducted before any modeling and was found to yield excellent covariate balance (Figure 2A). Over a median follow-up time of 46.8 months (interquartile range: 21.1, 80.7), in a multivariable IPTW Cox regression model, the presence of a baseline mental health diagnosis was associated with a 14% increased risk of all-cause mortality (HR=1.14; 95% CI: 1.12–1.15; *p* < 0.001) (Table 2, model 1). With the addition of time-updated mental health comorbidity every 30 days to the model (to account for incident mental health diagnoses during follow-up), the presence of any mental health diagnosis was associated with a 54% increased hazard in all-cause mortality (HR=1.54; 95% CI: 1.52–1.56; *p* < 0.001) (Table 2, model 2 and Figure 3A). Results were essentially unchanged in a sensitivity analysis where CIRCOM scores were incorporated as an additional covariate. For example, in the model 1 analysis the HR was 1.14 (1.12–1.15, *p* < 0.001), and in the model 2 analysis the HR was 1.57 (1.54–1.60, *p* < 0.001). In subgroup analysis limited to patients with any time-updating mental health diagnosis, regular outpatient mental health care was associated with a 3% reduction in all-cause mortality (HR=0.97; 95% CI: 0.95–0.99; *p* < 0.001) (Table 2, model 3). In a sensitivity analysis limited to patients who had at least 2 outpatient visits in the index year, results from the time-updated mental health comorbidity model (HR=1.55; 95% CI: 1.53–1.57; *p* < 0.001) and those from the subgroup analysis were essentially unchanged (HR=0.97; 95% CI: 0.96–0.99; *p*=0.002).

**TABLE 2:** Inverse probability treatment weighting–adjusted Cox regression models for all-cause mortality with any MH diagnosis as primary exposure (S1)

	Model 1: Baseline MH diagnosis		Model 2: Time-updating MH diagnosis		Model 3: Subgroup analysis of patients with time-updating MH diagnosis	
	HR (95% CI)	*p*	HR (95% CI)	*p*	HR (95% CI)	*p*
Any MH diagnosis	1.14 (1.12–1.15)	<0.001	1.54 (1.52–1.56)	<0.001	—	—
Regular MH visits (any)	—	—	—	—	0.97 (0.95–0.99)	<0.001

Abbreviation: MH, mental health.

### Association of non-AUD/SUD diagnosis with all-cause mortality

In a similar manner before modeling, IPTW adjustment based on PS was used for the non-AUD/SUD category. The excluded AUD and SUD comorbidities were additionally incorporated into the IPTW to ensure balance in these conditions and demonstrated superb covariate balance (Figure 2B). In IPTW Cox regression, baseline non-AUD/SUD was associated with a 4% increased hazard of all-cause mortality (HR=1.04; 95% CI: 1.03–1.05; *p* < 0.001) (Table 3, model 1). In the time-updated mental health comorbidity model, non-AUD/SUD diagnosis was associated with an 11% increased risk in all-cause mortality (HR=1.11; 95% CI: 1.10–1.13; *p* < 0.001) (Table 3, model 2 and Figure 3B). Further evaluating the protective factor of regular mental health visits with the time-updated mental health comorbidity revealed a 9% decreased hazard in all-cause mortality (HR=0.91; 95% CI: 0.90–0.93; *p* < 0.001) (Table 3, model 3).

**TABLE 3:** Inverse probability treatment weighting–adjusted Cox regression models for all-cause mortality with non-AUD/SUD MH diagnoses as primary exposure (S2)

	Model 1: Baseline non-AUD/SUD MH diagnosis		Model 2^a^: Time-updating non-AUD/SUD MH diagnosis		Model 3^a^: Subgroup analysis of patients with time-updating non-AUD/SUD MH diagnosis	
	HR (95% CI)	*p*	HR (95% CI)	*p*	HR (95% CI)	*p*
Any non-AUD/SUD MH diagnosis	1.04 (1.03–1.05)	<0.001	1.11 (1.10–1.13)	<0.001	—	—
Regular non-AUD/SUD MH outpatient visits	—	—	—	—	0.91 (0.90–0.93)	<0.001

^a^ Model also adjusted for time-updating alcohol use disorder and substance use disorder diagnoses.

Abbreviations: AUD, alcohol use disorder; MH, mental health; SUD, substance use disorder.

### Association of AUD/SUD diagnosis with all-cause mortality

Again IPTW adjustment based on PS was used for the AUD/SUD category, and the excluded diagnoses of depression, anxiety disorder, PTSD, bipolar disorder, and schizophrenia were incorporated into the IPTW with outstanding covariate balance (Figure 2C). Using IPTW Cox regression model, baseline AUD/SUD was associated with a 15% increased hazard of all-cause mortality (HR=1.15; 95% CI: 1.14–1.17; *p* < 0.001) (Table 4, model 1). With the addition of time-updated variable to the model, AUD/SUD diagnosis was associated with a 44% increased risk in all-cause mortality (HR=1.44; 95% CI: 1.42–1.46; *p* < 0.001) (Table 4, model 2 and Figure 3C). Any regular mental health visit revealed a 21% decreased risk in all-cause mortality (HR=0.79; 95% CI: 0.77–0.83; *p* < 0.001) (Table 4, model 3).

**TABLE 4:** Inverse probability treatment weighting–adjusted Cox regression models for all-cause mortality with AUD/SUD MH diagnoses as primary exposure (S3)

	Model 1: Baseline AUD/SUD MH diagnosis		Model 2^a^: Time-updating AUD/SUD MH diagnosis		Model 3^a^: Subgroup analysis of patients with time-updating AUD/SUD MH diagnosis	
	HR (95% CI)	*p*	HR (95% CI)	*p*	HR (95% CI)	*p*
Any AUD/SUD diagnosis	1.15 (1.14–1.17)	<0.001	1.44 (1.42–1.46)	<0.001	—	—
Regular AUD/SUD MH outpatient visits	—	—	—	—	0.79 (0.77–0.82)	<0.001

^a^ Model also adjusted for time-updating depression, anxiety disorder, posttraumatic stress disorder, bipolar disorder, and schizophrenia diagnoses.

Abbreviations: AUD, alcohol use disorder; MH, mental health; SUD, substance use disorder.

### Exploratory analysis

In cause-specific mortality models exploring the impact of any time-updated mental health comorbidity on liver-related or non–liver-related mortality, mental health comorbidities were associated with an increased hazard of both types of mortality relative to patients with no mental health comorbidities. However, the effect size was larger for liver-related mortality; the cause-specific HR for non–liver-related mortality was 1.36 (95% CI: 1.33–1.40, *p* < 0.001) and for liver-related mortality was 1.62 (95% CI: 1.59–1.65, *p* < 0.001).

## DISCUSSION

In this large national analysis of VHA patients with cirrhosis, we identified an association between the presence of mental health disease and increased hazard of all-cause mortality. Moreover, among specific types of diagnoses (ie, non-AUD/SUD diagnosis vs. AUD/SUD diagnosis), those with AUD or SUD had the highest risk of all-cause mortality. Importantly, we found that regular outpatient mental health care was associated with reduced all-cause mortality for patients with any mental health diagnosis, and this effect was particularly salient for patients with AUD or SUD.

The literature surrounding the association of mental health disease with mortality in patients with cirrhosis supports our findings but is rather limited in both scope and size. The few identified studies show a similar association of mental health disease with increased mortality in patients with liver disease with a mostly limited focus on depression and/or anxiety alone.^5^,^6^ More robust literature in other chronic conditions (eg, diabetes mellitus, asthma, heart failure) can be extrapolated to the CLD population and similarly demonstrates an association between comorbid mental illness and worsened health outcomes, such as reduced quality of life, increased frailty, and increased mortality.^4^,^33^

Large studies addressing the impact of diverse mental health conditions in patients with cirrhosis, particularly in the veteran population that is enriched in mental health comorbidities, have remained a gap in the literature. An additional important distinguishing feature of our study is the concerted effort to account for not only baseline confounders through IPTW, but also for time-updated covariates in the models. This enabled us to evaluate the importance of one mental health comorbidity category while systematically controlling for the other. In these analyses, our results consistently demonstrated the significant association between comorbid mental illness and increased all-cause mortality, inclusive of models with AUD/SUD diagnosis, non-AUD/SUD diagnosis, and both AUD/SUD and non-AUD/SUD diagnoses. Some proposed mediators of this association include: reduced patient engagement in care, stigmatization altering patient and provider interactions, reduced adherence to routine cirrhosis-related screening, medication nonadherence, engagement in risky behaviors, self-injury habits, reduced self-care (including poor nutrition, physical inactivity, and poor sleep), and decreased likelihood of liver transplant consideration. In our exploratory analysis with cause-specific mortality models, we found that any mental health diagnosis was associated with an increased hazard of both liver-related and non–liver-related mortality, however, this effect was more profound with liver-related mortality. This would suggest that some of the most salient mediators of increased mortality may be related to liver-specific care and preventative measures (eg, HCC surveillance or esophageal varices surveillance). Though this list of mediators is not all-encompassing and should be further explored in future studies, our findings that routine outpatient mental health care is associated with reduced all-cause mortality would be consistent with this conceptual model through mitigation of the above-mentioned mechanisms.

The trends in mental health diagnosis and outpatient care utilization identified in this VHA cirrhosis study are generally consistent with those national data from more general cohorts, and these trends may help to identify important care gaps in the status quo. The incidence of all individual mental health diagnoses in this study has been rising steadily since 2016. National data similarly demonstrate a rising burden of depression and anxiety in recent years, particularly among young adults less than 35 years of age.^34^,^35^ Over the course of the COVID-19 pandemic, several studies have also observed an increase in depression and anxiety in US adults compared with prepandemic levels.^36^,^37^ In regards to substance use, our study identified a recent rising incidence of AUD and SUD, which also mirrors national trends.^38^,^39^ A recent cross-sectional analysis by Desai et al^40^ evaluating US hospitalization trends found a rise in comorbid CLD-SUD hospitalizations resulting in higher rates of inpatient mortality than CLD alone, and during the COVID-19 pandemic there has been a rise in alcohol-associated hepatitis waitlistings and liver transplantation.^41^ While we observed a parallel increase in the number of general mental health clinic visits per year in line with the rising incidence proportion of broad mental health diagnoses, there was a gradual decline in overall AUD/SUD-related visits despite a recent rising incidence proportion of AUD/SUD. This diverging trend has also been observed in national data from the National Survey on Drug Use and Health specifically from 2017 to 2018 and 2019 to 2020,^42^ and suggests a potential mismatch in recognition and allocation of AUD/SUD-related resources. A recent retrospective cohort study using VHA data from 2011 to 2015 found that while behavioral and/or pharmacotherapy for AUD in patients with cirrhosis and AUD was associated with lower rates of hepatic decompensation and mortality, a startlingly low proportion of eligible patients received these therapies (behavioral 12%, pharmacological 0.4%, and both 1%).^14^ Our exploratory trends analysis suggests that both mental health diagnoses and outpatient care utilization vary substantially across etiologies of liver disease, highlighting one potential factor that may help improve allocation of mental health resources to patients in need. In particular, patients with HCV had a rising prevalence of AUD/SUD over time, but declining outpatient utilization of AUD/SUD-related visits, in contrast to ALD patients that had rising prevalence and visit rates over time. This suggests that the need for outpatient AUD/SUD-related treatment may be underrecognized in many patients with HCV cirrhosis. Though speculative, it is possible that the emergence of direct-acting antiviral agents has to some degree reduced clinical vigilance in these patients after widespread HCV cure.

Acknowledging the rising rates of mental illness in patients with cirrhosis and its association with mortality requires us to better understand practical and effective interventions to reduce these outcomes. One widely accepted intervention in this context is an integrated care approach that involves integrated liver care with mental health interventions and social services. Earlier studies before 2015 demonstrated success with this approach in patients with HCV with a particular focus on substance use.^4^,^43^,^44^ For example, 1 randomized controlled study found that integrating counseling and case management into routine HCV care for patients with comorbid HCV, SUD, and mental illness resulted in increased eligibility for HCV treatment,^43^ while another study found that integrating mental health interventions and case management into HCV clinics resulted in higher rates of antiviral initiation and sustained virologic response.^44^ More recently, several studies have detailed effective integrated care models mainly in the context of AUD/ALD in both the clinic and hospital setting. Fomin et al^45^ describe the creation of a pilot inpatient alcohol liver service consisting of a nurse practitioner and hepatologist that evaluate high-risk patients with AUD for underlying liver disease and refer patients to liver clinic if advanced ALD is detected. They found that patients who engaged with this inpatient team had higher rates of outpatient follow-up compared with those referred openly (44.1% compared with 27.0%), and all completed hepatitis A and B vaccination series.^45^ Within the transplant context, Addolorato et al^46^ showed that the presence of an alcohol addiction unit (staffed with experts in AUD) within the liver transplant center reduced alcohol relapse after liver transplant. Winder et al^47^ discussed the implementation and 1 year operation of a novel multidisciplinary ALD clinic that included transplant hepatologists, addiction psychologists, consultation-liaison psychiatrists, social workers, and hepatology nurses, for which longitudinal outcomes data are still emerging. Reflecting the promising results from the above studies, the 2019 American Association for the Study of Liver Diseases guidance on ALD specifically recommends multidisciplinary, integrated management of ALD and AUD.^48^ This is a critical step in recognizing that ALD cirrhosis is in fact a consequence of a primary psychiatric condition that requires medical and psychosocial collaborative management as the standard of care.

While the knowledge in this field continues to grow, it is important to recognize areas of needed attention both at the provider and system levels. Providers must be aware of the role psychological comorbidity plays in impacting overall mortality in patients with cirrhosis, and evidence-based tools should be implemented to effectively screen for mental health diagnoses in these patients. At the system level, additional studies are needed to elucidate barriers to engagement in regular mental health care and to identify avenues that improve accessibility and effectivity of mental health resources. Integrated care programs may be that very avenue, but there is a need for additional quality randomized controlled trials investigating the impact of integrated care models in patients with cirrhosis (of varying etiology) and concomitant mental illness, including non-SUD/AUD and AUD/SUD. Furthermore, unique articles such as the one published by Winder and colleagues that details the implementation of an integrated clinic program are needed to help lay the groundwork for future programs. Last, the realistic challenges in clinical practice to starting and successfully implementing integrated care programs that is extensively discussed by DiMartini et al^49^ must be further explored. We believe that the veteran population is the optimal population to engage in answering some of the above-mentioned questions and pilot integrated care programs due to the recognized high rates of mental health comorbidities and perhaps fewer system-level barriers such as inadequate mental health resources and absence of insurance coverage.

There are important limitations to acknowledge in this study. First, in models evaluating impact of regular outpatient mental health care visit, there is the possibility of a healthy user bias (ie, that patients who engage with regular mental health care are more likely to engage with general medical and cirrhosis care). However, in a sensitivity analysis limited to patients with at least outpatient visits in the index year, primary model results were similar. Second, misclassification of the primary exposures of mental health diagnoses and mental health care visits using ICD-9/10 codes and stop codes, respectively, could bias study results. To minimize this, we used classification methods supported by the literature wherever possible. In addition, if we assume that misclassification of our exposure variables led to underdetection of mental health comorbidities, our results would be biased towards the null hypothesis and therefore conservative. However, it is important to specifically note that classification of etiology of liver disease in this study, though based on a VHA validated algorithm, does not fully capture hazardous alcohol use a contributor to liver disease for many patients categorized as having non-ALD-related cirrhosis. This is supported by a recent observational study by Staufer et al^50^ demonstrating that nearly one third of patients with NAFLD have relevant alcohol intake to the degree of possibly inducing liver damage. Third, the veteran cohort used in this study is primarily male and with high rates of mental health comorbidities that may limit the generalizability of these results to other populations. Fourth, though our goal was to study the broad impacts of mental health diagnoses in our VHA cohort, we acknowledge that substantial heterogeneity exists within the mental health subcategories included in this study. For example, patients with schizophrenia or bipolar disorder were grouped together with patients with anxiety disorder or depression under the non-AUD/SUD category. We recognize that these disorders have vastly different clinical manifestations that could affect treatment engagement, adherence, and ultimately outcomes. Future studies should explore relevant differences in outcomes related to more specific mental health diagnoses. Fifth, although we used advanced inferential methods to address confounding including IPTW and time-updated covariate adjustment, there is always the possibility of residual confounding. Conclusions in this study should do not be regarded as demonstrating causal relationships but rather should be viewed as hypothesis generating. Sixth, we recognize the likelihood of heterogeneity among the SUD group (eg, opiate use vs. stimulant use vs. alcohol use, etc.) that we were not able to delineate in our analyses. Outcomes may change based on subcategories of substance use and this topic should be the basis for future investigation. Finally, our study was limited to the primary outcome of all-cause mortality. We did not explore other outcomes variables, such as cirrhosis decompensation, incidence of HCC diagnosis, or frequency of hospitalizations. This study lays the groundwork for future studies to expand on relevant outcome measures.

In summary, in this large cohort of veterans with cirrhosis, we found that mental health comorbidities were associated with increased all-cause mortality. Regular engagement with outpatient mental health care mitigated the risk of mortality, which was most profound in patients with AUD or SUD. These findings suggest that greater attention should be placed on recognizing psychological comorbidity in patients with cirrhosis and improved efforts should be made to facilitate patient engagement with mental health treatment, particularly in those struggling with substance use. This calls for a fundamental shift in our approach to cirrhosis care by which multidisciplinary integrated care models are at the center of patient care. Future studies should focus on elucidating the mechanisms underpinning the increased mortality observed in these patients, and both systems-level and provider-level changes that may impact these outcomes.

## Supplementary Material
