# Supplementary material for: The association between mental illness and all-cause mortality in patients with cirrhosis: a Veterans Affairs retrospective cohort study
Source: Hepatol Commun. 2023 Mar 30;7(4):e0129. doi: 10.1097/HC9.0000000000000129 (PMC10069831; doi:10.1097/HC9.0000000000000129)
Supplement: Supplementary file 1 [file hc9-7-e0129-s001.docx]

**Supplemental Table 1 - ICD-9/ICD-10 Codes Used to Identify Mental Health-Related Diagnoses**

| **Condition** | **ICD-9 codes** | **ICD-10 codes** |
| --- | --- | --- |
| Depression | 296.2, 296.3, 298.0, 300.4, 311 | F32, F33, F34 |
| Anxiety disorder | 300.0, 300.2 | F40, F41 |
| PTSD | 309.81 | F43.10, F43.12 |
| Bipolar disorder | 296.0, 296.1, 296.04, 296.14, 296.4, 296.44, 296.5, 296.44, 296.5, 296.54, 296.6, 296.7, 296.8 | F31 |
| Schizophrenia | 295.x | F20.x, F25 |
| Alcohol use disorder | 303.0, 303.9, 305.0 | F10.1, F10.2, F10.3 |
| Substance use disorder (except alcohol) | 304.x–305.x | F11.x–19.1-2, except F17 |

**Supplemental Table 2 – Stop Codes Used to Identify Outpatient Mental Health-Related Visits**

| **Stop Code** | **Description** | **Sub-Category** |
| --- | --- | --- |
| 156 | Home based primary care (HBPC)-psychologist | Mental health clinic/primary care |
| 157 | HBPC-psychiatrist | Mental health clinic/primary care |
| 323 | Mental health medical primary care | Mental health clinic/primary care |
| 338 | Telephone mental health medical primary care | Mental health clinic/primary care |
| 502 | Mental health clinic individual | Mental health clinic/primary care |
| 527 | Mental health telephone | Mental health clinic/primary care |
| 539 | Mental health integrated care group | Mental health clinic/primary care |
| 550 | Mental health clinic-group | Mental health clinic/primary care |
| 513 | Substance use disorder-individual | AUD/SUD |
| 514 | Substance use disorder-home visit | AUD/SUD |
| 519 | Substance use disorder/post-traumatic stress disorder (PTSD) teams | AUD/SUD |
| 523 | Opioid substitution | AUD/SUD |
| 545 | Telephone/substance use disorder | AUD/SUD |
| 547 | Intensive substance use disorder-group | AUD/SUD |
| 548 | Intensive substance use disorder-individual | AUD/SUD |
| 560 | Substance use disorder-group | AUD/SUD |
| 292 | Observation psychiatry | Other |
| 509 | Psychiatry-individual | Other |
| 510 | Psychology-individual | Other |
| 512 | Mental health consultation | Other |
| 516 | PTSD-group | Other |
| 528 | Telephone/homeless chronically mentally ill | Other |
| 532 | Psychosocial rehabilitation-individual | Other |
| 535 | MH vocational assistance-individual | Other |
| 536 | Telephone/MH vocational assistance | Other |
| 537 | Telephone/psychosocial rehabilitation | Other |
| 538 | Psychological testing | Other |
| 540 | PTSD clinical team (PCT) post-traumatic stress-individual | Other |
| 542 | Telephone/PTSD | Other |
| 546 | Telephone/ Mental health intensive case management (MHICM) | Other |
| 552 | MHICM | Other |
| 553 | Day treatment-group | Other |
| 554 | Day hospital-group | Other |
| 557 | Psychiatry-group | Other |
| 558 | Psychology-group | Other |
| 559 | Psychosocial rehab-group | Other |
| 562 | PTSD-individual | Other |
| 580 | PTSD Day Hospital | Other |
| 564 | Mental health team case management | Other |
| 565 | Mental health intervention biomed care group | Other |
| 566 | Mental health risk factor reduction emergency department group | Other |
| 567 | Mental health intensive case management group | Other |
| 568 | Mental health compensated work therapy/supported employment face to face | Other |
| 571 | Services for Returning Veterans- Mental Health (SeRV-MH)-individual | Other |
| 572 | SeRV-MH-group | Other |
| 573 | Mental health incentive therapy face-to-face | Other |
| 574 | Mental health compensated work therapy/transitional work experience (CWT/TWE) face-to-face | Other |
| 575 | Mental health vocational assistance group | Other |
| 582 | Psychosocial rehabilitation and recovery center (PRRC)-individual | Other |
| 583 | PRRC-group | Other |
| 584 | Telephone PRRC | Other |

**Supplemental Table 3 – Baseline Cohort Characteristics, Stratified by Presence/Absence of Any Mental Health Diagnosis**

| **Factor** | **No Mental Health Diagnosis**  **(N=21,131)** | **Mental Health Diagnosis**  **(N=94,278)** | **p-value** |
| --- | --- | --- | --- |
| Age, median (IQR) | 66 (60, 73) | 62 (57, 67) | <0.001 |
| Sex | 20641 (97.7%) | 91284 (96.8%) | <0.001 |
| Race |  |  | <0.001 |
| White | 14162 (67.0%) | 57268 (60.7%) |  |
| Black | 2730 (12.9%) | 18471 (19.6%) |  |
| Hispanic | 1710 (8.1%) | 7645 (8.1%) |  |
| Asian | 341 (1.6%) | 1178 (1.2%) |  |
| Other | 2188 (10.4%) | 9716 (10.3%) |  |
| BMI, median (IQR) | 29.6 (26.0, 34.0) | 28.3 (24.6, 32.7) | <0.001 |
| Etiology of Liver Disease |  |  | <0.001 |
| HCV | 4083 (19.4%) | 18053 (19.2%) |  |
| HBV | 341 (1.6%) | 797 (0.8%) |  |
| ALD | 4160 (19.8%) | 33686 (35.8%) |  |
| HCV+ALD | 1449 (6.9%) | 21056 (22.3%) |  |
| NAFLD | 8758 (41.7%) | 18723 (19.9%) |  |
| Other | 2222 (10.6%) | 1902 (2.0%) |  |
| Diabetes Mellitus | 11414 (54.0%) | 49564 (52.6%) | <0.001 |
| Coronary Artery Disease | 5308 (25.1%) | 24372 (25.9%) | 0.03 |
| Heart Failure | 3616 (17.1%) | 15429 (16.4%) | 0.008 |
| Atrial Fibrillation | 3019 (14.3%) | 10723 (11.4%) | <0.001 |
| CTP Class |  |  | <0.001 |
| A | 13300 (62.9%) | 59212 (62.8%) |  |
| B | 6725 (31.8%) | 29316 (31.1%) |  |
| C | 1106 (5.2%) | 5750 (6.1%) |  |
| Decompensated Cirrhosis | 4875 (23.1%) | 21427 (22.7%) | 0.28 |
| TIPS | 22 (0.1%) | 79 (0.1%) | 0.37 |
| Sodium, median (IQR) | 138 (136, 140) | 138 (135, 140) | <0.001 |
| Creatinine, median (IQR) | 1.0 (0.8, 1.4) | 0.9 (0.8, 1.2) | <0.001 |
| Albumin, median (IQR) | 3.5 (3.0, 4.0) | 3.5 (2.9, 4.0) | <0.001 |
| Total Bilirubin, median (IQR) | 1.0 (0.7, 1.7) | 0.9 (0.6, 1.6) | <0.001 |
| Alk Phos, median (IQR) | 98 (73, 140) | 102 (76, 143) | <0.001 |
| AST, median (IQR) | 45 (29, 72) | 52 (31, 91) | <0.001 |
| ALT, median (IQR) | 36 (23, 62) | 39 (24, 68) | <0.001 |
| Platelet Count, median (IQR) | 131 (92, 181) | 142 (100, 197) | <0.001 |
| INR, median (IQR) | 1.2 (1.1, 1.4) | 1.2 (1.1, 1.4) | <0.001 |
| MELD-Na, median (IQR) | 10 (6, 16) | 10 (6, 15) | 0.38 |
| HLD, median (IQR) | 40 (32, 52) | 41 (31, 54) | <0.001 |
| TGL, median (IQR) | 102 (73, 149) | 108 (77, 158) | <0.001 |
| LDL, median (IQR) | 80 (60, 103) | 82 (62, 107) | <0.001 |
| T. Cholesterol, median (IQR) | 146 (120, 174) | 150 (124, 180) | <0.001 |

*Abbreviations: MH = mental health, HCV = hepatitis C virus, HBV = hepatitis B virus, ALD = alcohol-related liver disease, NAFLD = non-alcoholic fatty liver disease, CTP = Child-Turcotte-Pugh*

**Supplemental Figure 1: Cohort Flow Diagram**


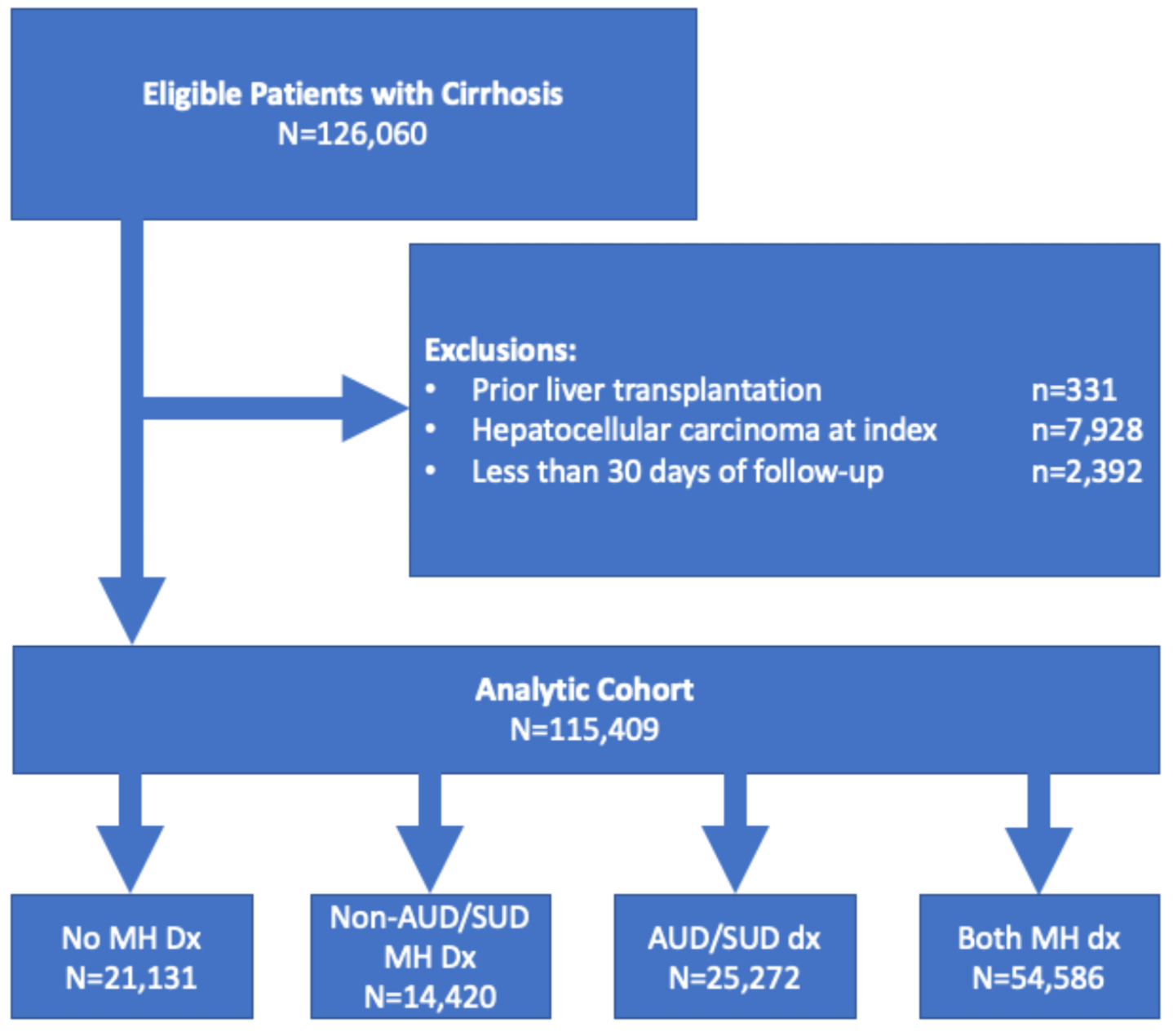


**Supplemental Figure 2 - Trends in Incidence Proportion of Mental Health Diagnoses**


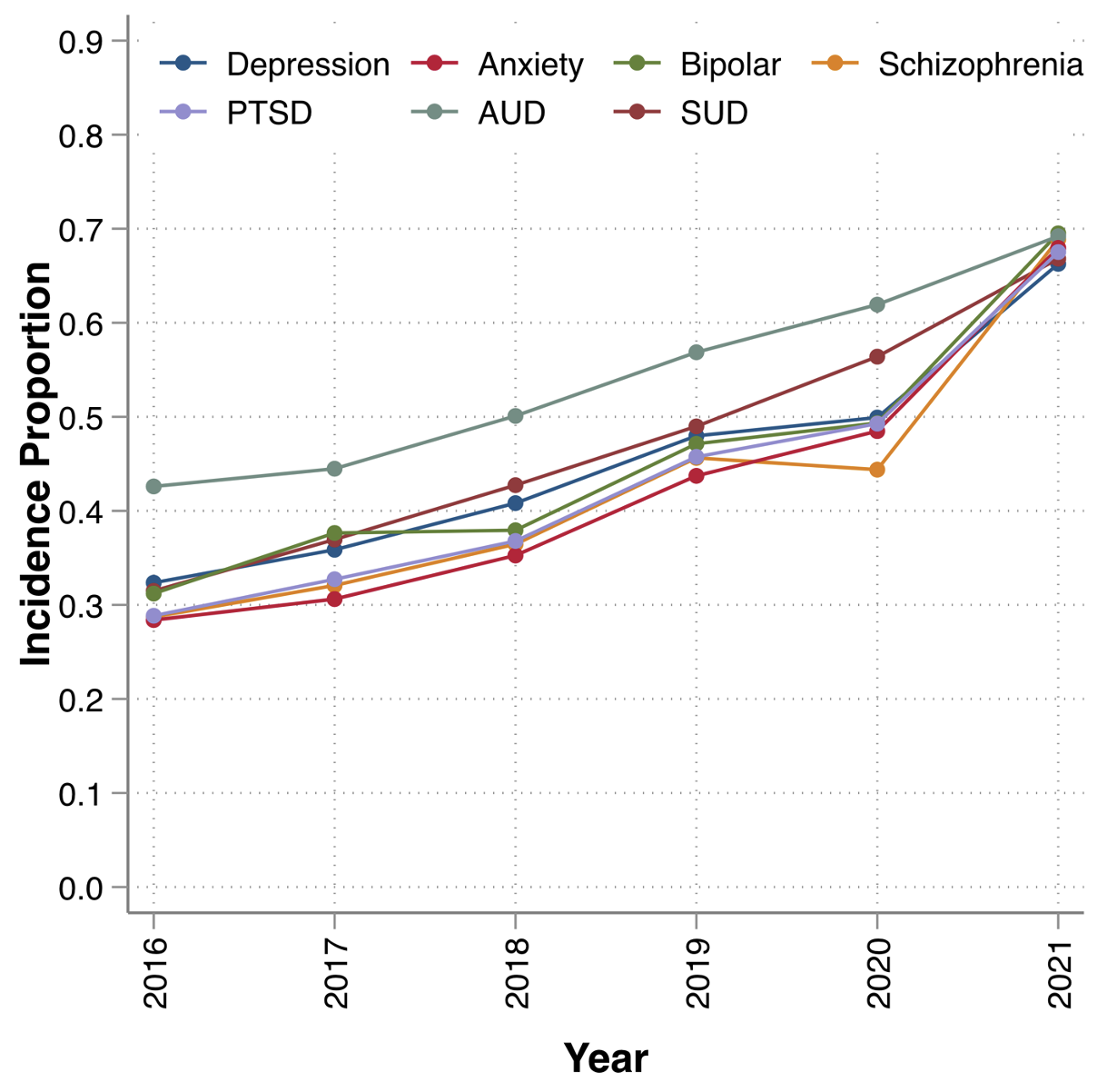


**Supplemental Figure 3 - Trends in Prevalence of Mental Health Diagnoses and Utilization of Outpatient Mental Health Care in Patients with Hepatis C Virus-related Cirrhosis (panels A/B), Non-alcoholic Fatty Liver Disease-related Cirrhosis (panels C/D), and Alcohol-related Liver Disease (panel E/F)**
